# Off-label use of clonidine for sedation in Dutch ICUs

**DOI:** 10.1186/cc13605

**Published:** 2014-03-17

**Authors:** J Van der Valk, M Zeeman, M Arbouw, H Van den Oever

**Affiliations:** 1 Deventer Hospital, Deventer, the Netherlands

## Introduction

Clonidine is an α_2_-agonist, licensed in most countries for treatment of hypertension. Other pharmacologic characteristics of α_2_-agonists are sedation, hypnosis, anxiolysis, sympatholysis, and analgesia [1]. Because of these central nervous effects, clonidine can be used off-label to augment sedation and delirium treatment. To our knowledge there have been no publications evaluating the efficacy and safety of clonidine in ventilated critically ill adults. A survey in German ICUs showed that clonidine was used for sedation in 62% of units [2]. We undertook an enquiry to investigate the off-label use of clonidine in Dutch ICUs.

## Methods

An inventory was sent by email to 25 ICUs in the Netherlands, determined by the difficulty to identify a contact person.

## Results

The response rate was 56%. The results are summarized in Table 1. Clonidine was used in all 14 responding ICUs; in 36% this use was reported as often. Licensed use (for hypertension) was 50%. Indications for off-label use were: substance withdrawal (50%), delirium (71%), and sedation (29%). The route of administration was intravenous in all cases. Nine ICUs reported the use of a loading dose, irrespective of indication, varying from 10 to 150 μg, median 150 μg. Ten ICUs reported the use of continuous infusion, varying from 10 to 100 μg/ hour, median 50 μg/hour.

**Table 1:** Indication and doses of clonidine used in Dutch ICUs

IC level^a^	Clonidine use	Indication	Loading dose tμg)	Continuous infusion dose μ/hour)
3	Often	Hypertension sedation	ns	ns
3	Often	Hypertension withdrawal; hypertension delirium; sedatior	40 to 12C	10 to 80
3	Often	Substance withdrawal; sedation	150	50
3	Often	Substance withdrawal	75 to 150	50
3	Often	Substance withdrawal; hypertension; delirium	10	40 to 100
3	Sometimes	Delirium	ns	30 to 100
3	Sometimes	Hypertension; delirium	150	ns
3	Sometimes	Substance withdrawal; delirium	ns	40
2	Sometimes	Substance withdrawal; delirium	ns	20 to 50
2	Sometimes	Hypertension; sedation	150	40
2	Sometimes	Substance withdrawal; hypertension; delirium	150	12 to 25
1	Sometimes	Hypertension; delirium	150	ns
1	Sometimes	Delirium	50	50 to 100
1	Sometimes	Delirium	ns	ns

ns, not specified. ^a^Level of ICU care, with level 3 being the most advanced.

## Conclusion

Off-label use of clonidine for sedation and treatment of withdrawal symptoms and delirium was common practice in Dutch ICUs. There was a wide range of dosing regimens: infusion schedules varied 10-fold and loading doses 15-fold. Clinical studies are required to establish the safety and efficacy of clonidine in the ICU setting.
